# Effect of administration route and dose escalation on plasma and intestinal concentrations of enrofloxacin and ciprofloxacin in broiler chickens

**DOI:** 10.1186/s12917-014-0289-1

**Published:** 2014-12-02

**Authors:** Mathias Devreese, Gunther Antonissen, Siegrid De Baere, Patrick De Backer, Siska Croubels

**Affiliations:** Department of Pharmacology, Toxicology and Biochemistry, Faculty of Veterinary Medicine, Ghent University, Salisburylaan 133, 9820 Merelbeke, Belgium

**Keywords:** Enrofloxacin, Broiler chicken, Dose escalation, Route of administration, Plasma, Intestinal content

## Abstract

**Background:**

The (mis)use of fluoroquinolones in the fowl industry has led to an alarming incidence of fluoroquinolone resistance in pathogenic as well as commensal bacteria. Next to simply reducing antimicrobial consumption, optimizing dosage regimens can be regarded as a suitable strategy to reduce antimicrobial resistance development without jeopardizing therapy efficacy and outcome. A first step in order to limit antimicrobial resistance is to assess the exposure of the intestinal microbiota to enrofloxacin after different treatment strategies. Therefore, a study was conducted in broiler chickens to assess the effect of route of administration (oral versus intramuscular) and dose escalation (10 and 50 mg/kg body weight) on plasma and intestinal concentrations of enrofloxacin and its main metabolite ciprofloxacin after treatment with enrofloxacin once daily for five consecutive days. Four different parts of the intestinal tract were sampled: ileum, cecum, colon and cloaca. A liquid chromatography-tandem mass spectrometry (LC-MS/MS) method was developed to quantify both analytes in plasma and intestinal content. Sample preparation prior to LC-MS/MS analysis consisted of extraction with ethyl acetate. For intestinal content samples PBS buffer was added before extraction. The supernatant was evaporated to dryness and resuspended in water prior to analysis.

**Results:**

The results in plasma and intestinal content demonstrated that biotransformation of enro- to ciprofloxacin in broiler chickens is limited. In general, the intestinal microbiota in cecum and colon is exposed to significant levels of enrofloxacin after conventional treatment (21–130 μg/g). A clear increase of intestinal concentrations was demonstrated after administration of a five-fold higher dose (31–454 μg/g). After intramuscular administration, intestinal concentrations were comparable, except for the higher levels in cloaca due to the complete bioavailability and urinary excretion.

**Conclusions:**

The intestinal microbiota is exposed to high levels of the antimicrobial, after oral as well as parenteral therapy. Furthermore, a dose and time dependent correlation was observed. The impact of the detected intestinal levels on resistance selection in the intestinal microbiota has to be further investigated.

**Electronic supplementary material:**

The online version of this article (doi:10.1186/s12917-014-0289-1) contains supplementary material, which is available to authorized users.

## Background

Enrofloxacin is a fluoroquinolone chemotherapeutic frequently used in veterinary medicine. This broad spectrum antimicrobial is indicated in poultry for the treatment of respiratory and intestinal tract infections caused by *Mycoplasma gallisepticum*, *Mycoplasma synoviae*, *Avibacterium gallinarum*, *Pasteurella multocida* and *Escherichia coli (E. coli)*. The (mis)use of this class of drugs in the fowl industry has led to an alarming incidence of fluoroquinolone resistance in pathogenic as well as commensal bacteria [1]. According to a recent report by the European Food Safety Authority (EFSA), ciprofloxacin resistance in *Salmonella spp*., *Campylobacter jejuni*, *Campylobacter coli* and the indicator bacteria *E. coli* derived from domestic fowl was 37.3%, 44.1%, 78.4% and 57.6%, respectively [2]. Ciprofloxacin is used as representative for the fluoroquinolones as it is used in human medicine and it is also the major metabolite of enrofloxacin. Although in broiler chickens the metabolisation of enro- to ciprofloxacin is limited, 5 to 10% [3,4]. The high rate of fluoroquinolone resistance is not only of concern for veterinary medicine (e.g. treatment failure) but also for human medicine as resistant bacteria can be transferred through the food chain, through direct contact with animals or through the environment (contaminated soil) [5].

A clear association between antimicrobial drug use and appearance of antimicrobial resistance has been demonstrated [6,7]. These issues have led to an increasing awareness to reduce the use of critically important antimicrobials in intensively reared livestock, for example by prohibiting the use of antimicrobials as growth promoters in Europe since 2006 [8], the ban to use fluoroquinolones in the US since 2005 [9] and implementing control systems monitoring antimicrobial consumption in several European countries including the Netherlands (MARAN), Denmark (DANMAP) and Belgium (AMCRA).

Next to simply reducing antimicrobial consumption, optimizing dosage regimens can be regarded as a suitable strategy to reduce antimicrobial resistance development without jeopardizing therapy efficacy and outcome. The current posology of veterinary antimicrobial drugs is determined by dose titration and confirmation studies solely monitoring clinical efficacy. Limiting the emergence and spread of resistance is not taken into account in these studies. Haritova et al. (2011) [10] showed that a high dose of enrofloxacin given in a short time resulted in better eradication of pathogenic *E. coli* (O78/H12) in broiler chickens compared to the conventional treatment. Furthermore, it has been suggested that parenteral administration is preferred over oral administration as the intestinal commensal microbiota is less exposed to the antimicrobial leading to more limited resistance selection [11]. Nevertheless, the low economic value of individual birds makes parenteral therapy cost ineffective and drinking water is the preferred route to administer mass medication as sick birds continue to drink. Furthermore, some considerations have to be made with intramuscular administration to birds. Necrotic lesions in the pectoral muscles can occur after intramuscular injection in the breast muscle, hereby reducing the quality of the resulting meat [12]. Injection in the leg muscle on the other hand, might lower the bioavailability of the drug through direct elimination in the kidneys because of the renal-portal system [13]. Recently, the Scientific Committee of the Federal Agency for the Safety of the Food Chain (FASFC) of Belgium reported that the few studies evaluating the effect of oral versus parenteral therapy on resistance selection in the intestinal commensal microbiota are inconclusive [14]. In order to optimize the dosage strategy of fluoroquinolones in poultry, taking resistance selection of the intestinal microbiota into account, a first objective is to determine the antimicrobial exposure of the intestinal microbiota after different treatments.

Therefore, the aim of the present study was to assess and compare the plasma and intestinal concentration of enro- and ciprofloxacin in broilers treated with the conventional dosage regimen, broilers treated with an elevated dose and the effect of oral versus parental route of administration.

## Methods

### Chemicals, products and reagents

The analytical standards of enro-, cipro and sarafloxacin (internal standard, IS) were obtained from Sigma-Aldrich (Bornem, Belgium). Water, methanol and acetonitrile (ACN) were of LC-MS grade and obtained from Biosolve (Valkenswaard, The Netherlands). Glacial acetic acid and ethyl acetate were of analytical grade and obtained from VWR (Leuven, Belgium). Phosphate Buffered Saline (PBS) was obtained from Life Technologies (Gent, Belgium).

### Preparation of standard solutions

Separate standard stock solutions of enro-, cipro- and sarafloxacin were prepared in 0.01 M acetic acid in water (analyte concentration: 1 mg/mL) and were stored at ≤ −15°C. A working solution of sarafloxacin and a combined working solution of 0.1 mg/mL of enro- and ciprofloxacin was prepared by transferring 100 μL of each stock solution into an Eppendorf cup, followed by further dilution with water up to a final volume of 1.0 mL. Ten-fold dilutions were obtained by dilution with water. All working solutions were stored at 2-8°C.

### Animal experiment

Ninety-six three-week-old broiler chickens (Ross 308) of mixed gender were equally divided in 4 groups and allocated to a different treatment with enrofloxacin. Animals had *ad libitum* access to feed and drinking water throughout the experiment. After a one-week acclimatization period, the animals of the first group were administered the conventional treatment. Enrofloxacin (Baytril® 10 % oral solution, Bayer, Diegem, Belgium) was given as an oral bolus directly in the crop for 5 consecutive days (10 mg enrofloxacin/kg BW). The second group received an elevated dose of enrofloxacin orally (50 mg/kg BW) for five days. Enrofloxacin was given intramuscularly (*ad random* as several separate injections in both breast muscles) for 5 days at a dose of 10 or 50 mg/kg BW (Baytril® 5%, Bayer) to the birds of the other two groups.

From each group, eight animals were euthanized at each sampling point, namely 2 and 4 h after the first administration and 4 h after the last administration (100 h after first administration). Euthanasia was performed by sodium pentobarbital injection followed by exsanguination. Blood samples (1 mL) from the jugular vein and intestinal content from ileum, cecum, colon and cloaca were collected. Blood samples were centrifugated (2851 × *g*, 10 min, 4°C). Aliquots (250 μL) of plasma samples were stored at ≤ −15°C until analysis. Intestinal content samples of each treatment group were pooled by segment and stored at ≤ −80°C until analysis. This animal experiment was approved by the Ethical Committee of Ghent University (Case number EC 2013_117).

### Sample pretreatment

To 250 μL of plasma were added 12.5 μL of the IS working solution and vortex mixed (15 sec). Three mL of ethyl acetate were added, samples were extracted for 15 min on a roller mixer (Stuart Scientific, Surrey, UK) and centrifugated (2851 × *g*, 10 min, 4°C). Next, the supernatant was transferred to another tube and evaporated using a gentle nitrogen (N_2_) stream (45 ± 5°C). The dry residue was reconstituted in 250 μL of water. After vortex mixing (15 sec), the sample was transferred into an autosampler vial and an aliquot (5 μL) was injected onto the LC-MS/MS instrument.

To one gram of intestinal content, pooled per segment, were added 100 μL of the IS working solution followed by a vortex mixing step (15 sec) and addition of 3 mL PBS and 5 mL ethyl acetate. Samples were then treated in the same way as plasma samples. If the detected concentration was out of the linear range, samples were appropriately diluted with PBS, re-extracted and re-analyzed. Samples were analyzed in triplicate (technical replicates).

### Liquid chromatography

The LC system consisted of a quaternary, low-pressure mixing pump with vacuum degassing, type Surveyor MSpump Plus and an autosampler with temperature controlled tray and column oven, type Autosampler Plus, both from ThermoFisher Scientific (Breda, The Netherlands). Chromatographic separation was achieved on a Zorbax Eclipse Plus column (100 mm × 3.0 mm i.d., dp: 3.5 μm) in combination with a guard column of the same type (13 mm × 3.0 mm i.d., dp: 3.5 μm), both from Agilent (Diegem, Belgium). The temperatures of the column oven and autosampler tray were set a 45°C and 5°C, respectively. Mobile phase A consisted of 0.1% glacial acetic acid in water whereas mobile phase B was ACN. Following gradient elution program was run: 0–3.5 min (92% A, 8% B), 3.5-4.0 min (linear gradient to 80% A), 4.0-8.0 min (80% A, 20% B), 8.0-8.5 min (linear gradient to 92% A), 8.5-13.0 min (92% A, 8% B). Flow rate was set at 500 μL/min.

### Mass spectrometry and method validation

The LC column effluent was interfaced to a TSQ® Quantum Ultra triple quadrupole mass spectrometer, equipped with a heated electrospray ionization (h-ESI) probe operating in the positive ionization mode (all from ThermoFisher Scientific). Instrument parameters were optimized by syringe infusion of working solutions of 1 μg/mL of each compound (flow rate 10 μL/min) in combination with the mobile phases (50% A, 50% B).

The following general MS/MS parameters were used: spray voltage: 3800 V, vaporizer temperature: 300°C, sheath gas pressure: 33 au (arbitrary units), ion sweep gas pressure: 2.0 au, auxilliary gas pressure: 15 au, capillary temperature: 300°C, collision pressure: −1.5 mTorr and quad MS/MS bias: 2.9. The resolution for Q1 and Q3 were set at 0.7 peak width at half-height.

Acquisition was performed in the selected reaction monitoring (SRM) mode. For each compound, the two most intense product ions of the precursor ion were monitored in the SRM mode for quantification and identification, respectively. The SRM transitions for enro-, cipro and sarafloxacin were m/z 360.0 > 316.2*/245.1, 332.0 > 288.1*/314.1 and 386.1 > 299.0*/368.0, respectively. The * indicates the ion used for quantification.

The method was validated for enro- and ciprofloxacin in plasma as well as intestinal content according to a validation protocol previously described by De Baere et al. (2011) [15]. A set of parameters that were in compliance with the recommendations and guidelines defined by the European Community and with criteria described in the literature, were evaluated [16–18].

### Statistical analysis

After determination of normality and homogeneity of variances, one-way ANOVA (SPSS 20.0, IBM, Chicago, IL, USA) was performed on the results from the four different treatments within each matrix and sampling time point. A Scheffé test was performed as post-hoc test. The significance level was set at 0.05.

## Results

### LC-MS/MS method

Matrix-matched calibration graphs (1/x^2^ weighed) were linear over the working concentration range for enro- and ciprofloxacin in both plasma and intestinal content (see Additional file 1), with r values ranging between 0.9964 and 0.9988 and g values between 4.3% and 8.7%. The within- and between run accuracy and precision was evaluated at 2 or 3 concentration levels and fell within the acceptability ranges (see Additional file 2). The limits of quantification (LOQ) were 50 and 20 ng/mL for enro- and ciprofloxacin in plasma, respectively, whereas in intestinal content the LOQ was 100 ng/g. The limits of detection (LOD) varied between 0.07 and 1.19 ng/mL or ng/g (see Additional file 1). Matrix effects (signal suppression or enhancement, SSE) were determined and were 85.1% and 84.4% for enro- and ciprofloxacin in plasma, respectively, and 85.9% and 73.2% for enro- and ciprofloxacin in intestinal content, respectively. Moreover, this limited signal suppression was outweighed by the IS displaying a similar retention time.

### Animal experiment

**Figure 1:**
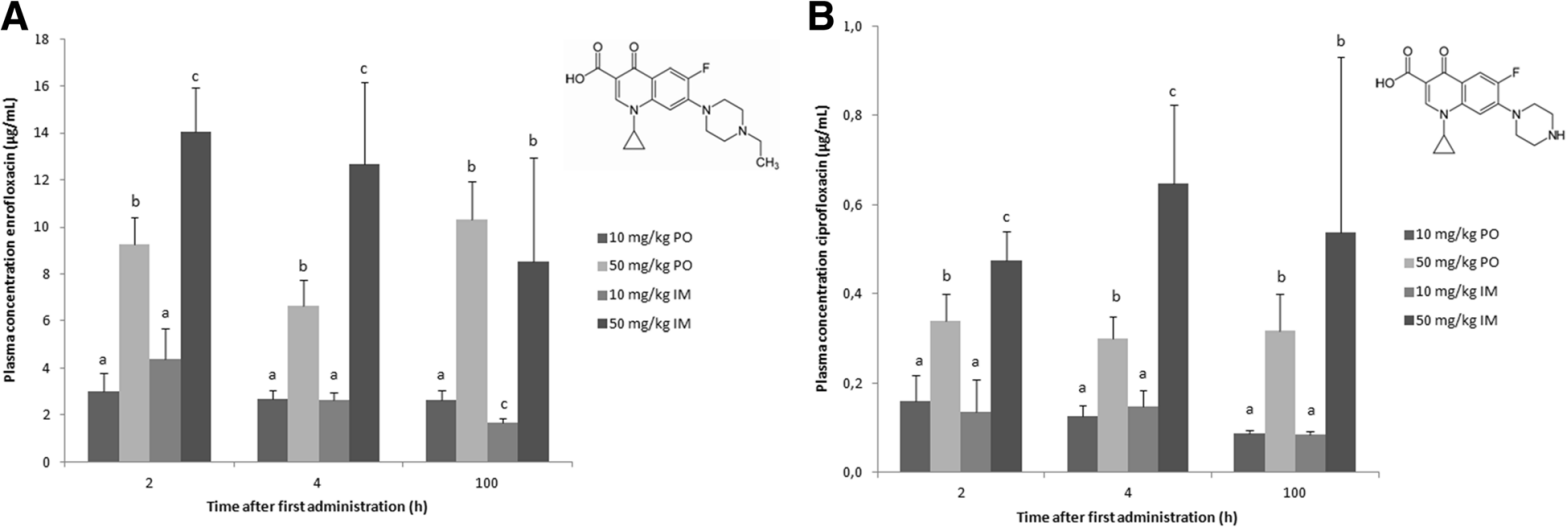
**Plasma concentrations (average + SD) of enro- (A) and ciprofloxacin (B) 2, 4 and 100 hours after the first oral (PO) or intramuscular (IM) administration of 10 or 50 mg enrofloxacin/kg body weight to broiler chickens given during 5 consecutive days (n = 8).** Values from the different treatment strategies with a different superscript within one time point, for the same compound (**(A)** or **(B)**), are statistically different at p < 0.05. The inserts show the chemical structure of enro- **(A)** and ciprofloxacin **(B)**.

The plasma concentrations of enro- and ciprofloxacin after oral or intramuscular administration are presented in Figure 1. Values present the mean (+ standard deviation, SD) of the 8 birds/group. Biotransformation of enro- to ciprofloxacin in broiler chickens is limited, as can be concluded from the concentration ranges measured for enrofloxacin: 1.66 to 14.04 μg/mL compared to ciprofloxacin: 0.08 to 0.65 μg/mL. The time of sampling, namely 2 or 4 h after first administration or 4 h after last administration (100 h), had a limited influence of the plasma levels of enro- and ciprofloxacin. However, dose escalation, from 10 mg/kg BW to 50 mg/kg BW, led to linearly increased plasma concentrations. At a dose of 10 mg/kg, the administration route (oral, PO, versus intramuscular, IM) altered the mean plasma concentrations only at 100 h (PO: 2.64 ± 0.42 μg/mL, IM: 1.66 ± 0.17 μg/mL) but not at 2 (PO: 3.02 ± 0.77 μg/mL, IM: 4.41 ± 1.26 μg/mL) and 4 h (PO: 2.69 ± 0.38 μg/mL, IM: 2.66 ± 0.31 μg/mL). In contrast, at the elevated dosage of 50 mg/kg, plasma levels at 2 (PO: 9.27 ± 1.15 μg/mL, IM: 14.04 ± 1.93 μg/mL) and 4 h (PO: 6.63 ± 1.12 μg/mL, IM: 12.66 ± 3.53 μg/mL) post administration were higher after parenteral administration, but not at 100 h (PO: 10.32 ± 1.62 μg/mL, IM: 8.54 ± 4.41 μg/mL).

**Figure 2:**
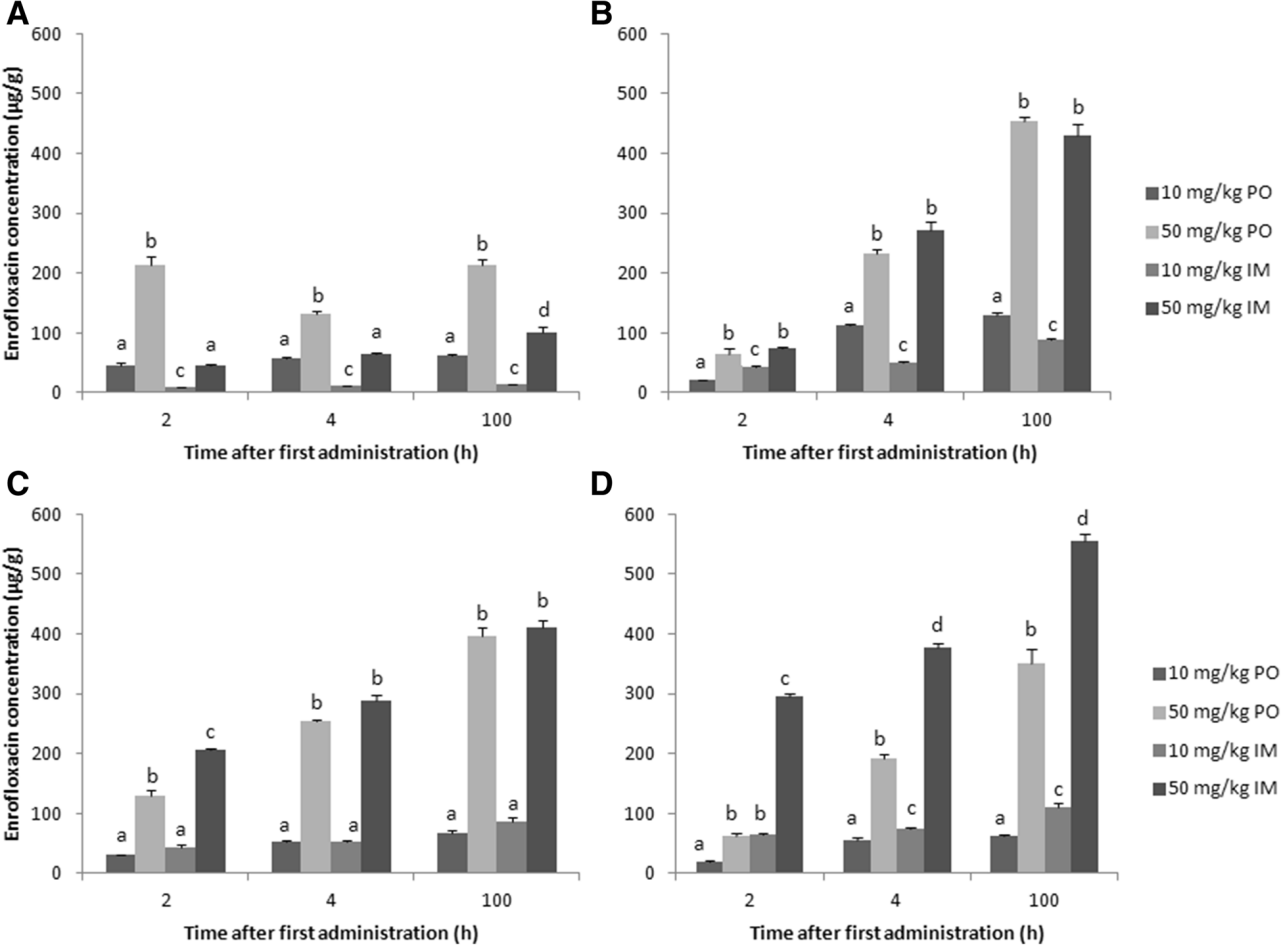
**Enrofloxacin concentrations (average + SD of the three technical replicates) in different parts of the intestinal tract content: ileum (A), cecum (B), colon (C) and cloaca (D), after oral (PO) or intramuscular (IM) administration of 10 mg enrofloxacin/kg BW or 50 mg enrofloxacin/kg BW a day for 5 consecutive days to broiler chickens.** Pooled samples (n = 8) were taken at 2, 4 and 100 h after first administration. Values from the different treatment strategies with a different superscript within one time point and within one intestinal segment are statistically different at p < 0.05.

**Figure 3:**
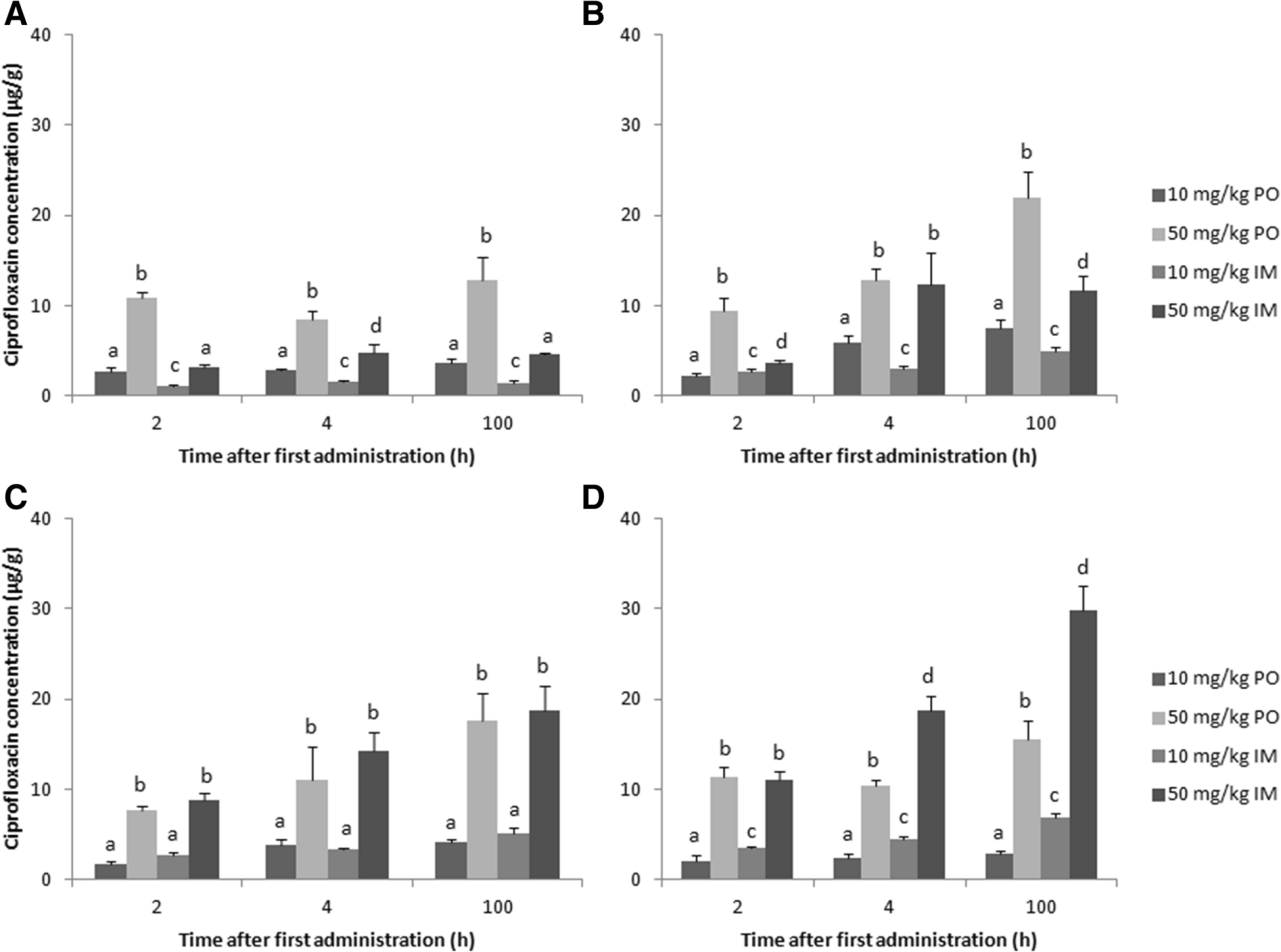
**Ciprofloxacin concentrations (average + SD of the three technical replicates) in different parts of the intestinal tract content: ileum (A), cecum (B), colon (C) and cloaca (D), after oral (PO) or intramuscular (IM) administration of 10 mg enrofloxacin/kg BW or 50 mg enrofloxacin/kg BW a day for 5 consecutive days to broiler chickens.** Pooled samples (n = 8) were taken at 2, 4 and 100 h after first administration. Values from the different treatment strategies with a different superscript within one time point and within one intestinal segment are statistically different at p < 0.05.

Intestinal concentrations of enro- and ciprofloxacin after treatment with enrofloxacin are presented in Figures 2 and 3, respectively. The values are presented as mean + SD of the three technical replicates from the pooled samples. Similar to the plasma results, the intestinal concentrations reported here indicate limited biotransformation of enro- to ciprofloxacin. Furthermore, a linear increase in enro- and ciprofloxacin levels after enrofloxacin dose escalation was observed. The route of administration had a limited effect on cecum and colon concentrations, whereas ileal and cloacal levels were respectively lower and higher after intramuscular administration at both doses used.

## Discussion

### LC-MS/MS method

This paper describes a validated LC-MS/MS method for the quantification of enrofloxacin, and its main metabolite ciprofloxacin in plasma and intestinal content of broiler chickens. Several methods for detection of fluoroquinolones in biological fluids have been described in literature. In the last decade, LC-MS/MS became the method of choice due to its high sensitivity and selectivity. Sample preparation generally exists of protein precipitation, solid-phase extraction (SPE) or liquid-liquid extraction. Protein precipitation is a cost effective and rapid procedure, however, matrix components can be enriched resulting in significant signal suppression or enhancement (SSE) or loss of sensitivity. SPE on the other hand, is generally time consuming and more expensive. A critical factor in the liquid-liquid extraction of amphoteric compounds such as fluoroquinolones is the neutralization of ionic compounds prior to extraction with e.g. ethyl acetate (pKa of carboxylic acid of enro- and ciprofloxacin: 6.0; pKa of the piperazine ring (amine functional group) of enro- and ciprofloxacin: 8.8 and 7.8 respectively) (Figure 1) [19]. To neutralize the variable pH of intestinal content effect, PBS was added prior to extraction. For plasma, a buffer was redundant as the pH is constant at ± 7.4. As expected, extraction with ethyl acetate in neutral conditions yielded the highest recoveries compared to acid or alkali conditions [20].

Chromatographic separation of fluoroquinolones is commonly performed by a combination of an organic solvent (ACN or methanol) and water with a volatile acid (e.g. acetic acid or formic acid) [21]. The most optimal chromatographic conditions were obtained with ACN in combination with 0.1% acetic acid in water. Concerning the MS/MS parameters, the highest sensitivity was obtained for the protonated parent compounds measured in the positive ESI mode and the followed SRM traces were in accordance with literature [19,21,22].

### Animal experiment

Current treatment strategies with antimicrobials exert a selective pressure not only on the pathogen where the treatment was intended for but also on the intestinal microbiota. To optimize dosage strategies and evaluate different administration routes, microbiota exposure data are mandatory. Therefore, an animal experiment was performed to assess and compare plasma and intestinal concentrations of enro- and ciprofloxacin after treatment of broiler chickens with the conventional dosage regimen (10 mg/kg BW), after a five-fold dose escalation and using different administration routes (oral and intramuscular).

Unlike in other animal species, biotransformation of enro- to ciprofloxacin is limited in poultry [3,23,24] and adds only minimally to the antimicrobial effect of enrofloxacin. At the T_max_ (2 h) after oral dosing the conventional treatment, mean maximum plasma concentration (C_max_) of enrofloxacin was 3.0 μg/mL, which is comparable to other literature reports [25]. The mean C_max_ of 4.4 μg/mL after 10 mg/kg BW intramuscular administration is also comparable to literature, where the C_max_ after 5 mg/kg BW IM was 2.1 μg/mL [20]. Furthermore, the increase of enro- and ciprofloxacin plasma concentrations after oral and intramuscular dose escalation was similar as described in broiler chickens and pigs by [10] and [26], respectively.

After oral bolus administration of enrofloxacin, a time dependent shift in enrofloxacin concentration was observed along the intestinal tract. Two hours after the first administration, the highest levels were detected in the ileum (10 mg/kg BW: 46.0 μg/g; 50 mg/kg BW: 214.9 μg/g), whereas four hours after first and last administration, the highest concentration were detected in the cecum (10 mg/kg BW: 112.1 μg/g after 4 h and 130.2 μg/g after 100 h; 50 mg/kg BW: 233.5 μg/g after 4 h and 453.6 μg/g after 100 h) and colon (10 mg/kg BW: 51.6 μg/g after 4 h, 67.2 μg/g after 100 h; 50 mg/kg BW: 254.9 μg/g after 4 h, 395.5 μg/g after 100 h). This corresponds with the fast transit time in fed chickens [27,28]. A similar time dependent shift of enrofloxacin from small to large intestine was already described in pigs [29]. As for plasma levels, a dose dependent correlation was observed for both oral and intramuscular administration. This corresponds with the results of Wiuff et al. (2003) [26] where an increase of the applied intramuscular dose led to elevated fecal concentrations in pigs (14.6 to 160.0 μg/g after administration of 2.5 to 15.0 mg/kg BW). In the present study, enro- and ciprofloxacin levels in the colon are comparable between oral and intramuscular administration: 30 – 67 and 44 – 86 μg enrofloxacin/g after 10 mg/kg BW PO and IM, respectively, and 1.8 – 4.1 and 2.6 – 5.2 μg ciprofloxacin/g after 10 mg/kg BW PO and IM, respectively. These high intestinal concentration after intramuscular administration may be attributed to biliary excretion and/or passive diffusion of fluoroquinolones based on their high volume of distribution (5.8 L/kg) and the relatively limited plasma protein binding (< 20%) [20,25,30]. Wiuff et al. (2002; 2003) [26,29] did not observe effects of administration route on fecal contents of enrofloxacin in pigs. In our study, cloacal concentrations after intramuscular administration (65 – 110 μg/g after 10 mg/kg BW) were higher compared to other intestinal segments. This can be attributed to the complete bioavailability after intramuscular administration compared to the moderate to high oral bioavailability (64.0% to 89.2%) [20,24,25] and to the fact that urine is the major route of excretion for fluoroquinolones. Recent reports [4,31] determined enrofloxacin concentration in poultry manure during and after oral treatment with enrofloxacin. In the study by Moraru et al. (2012) [31], broiler chickens were treated with 10 mg enrofloxacin/kg BW for 5 days and excreta samples were taken during and until 3 days post-administration. Detected concentrations ranged between 40.5 to 50.7 μg/g. A similar study [4] reported enrofloxacin concentrations in manure samples during treatment (15 mg enrofloxacin/kg BW, orally) ranging between 39.2 and 55.0 μg/g. In our study, cloacal concentrations ranged between 20.1 and 55.7 μg/g after oral treatment with 10 mg enrofloxacin/kg BW, which is comparable to the other reports.

In general, intestinal enrofloxacin concentrations detected in the present study are much higher compared to plasma concentrations. The ratio of enrofloxacin concentration in intestinal content/plasma of the four different treatment strategies ranges between 6.67 – 49.31, 6.59 – 43.96, 2.21 – 66.57 and 3.17 – 65.03 for treatment with 10 mg/kg BW PO, 50 mg/kg BW PO, 10 mg/kg BW IM and 50 mg/kg BW IM, respectively (see Additional file 3).

The next step is to correlate the obtained data with effects on resistance selection in the intestinal microbiota. This is done by correlating minimum inhibitory concentrations (MIC) with plasma pharmacokinetic characteristics, called pharmacokinetic-pharmacodynamic or PK-PD modelling. MIC values can be determined for pathogenic as well as commensal bacteria. MIC values for wild-type avian pathogenic *E. coli* (APEC) are variable and range from 0.016 to 0.5 μg/mL [1,10]. The breakpoint of C_max_/MIC ≥ 10 is an indicator for therapeutic efficacy of fluoroquinolones [32]. Hence, the conventional dosage regimen would reach this breakpoint with a MIC of 0.016 μg/mL (C_max_/MIC = 188) but not with a MIC of 0.5 μg/mL (C_max_/MIC = 6). With an elevated dose (50 mg/kg BW), the breakpoint value is reached even with the highest MIC. Intestinal enrofloxacin concentrations were considerably higher than plasma concentrations which would lead to an eradication of the pathogenic *E. coli* in the intestine for all treatment strategies. However, resistant commensal bacteria are more likely to transfer resistance from animals to man (e.g. fecal carcass contamination). For ciprofloxacin, the epidemiological cut-off value (ECOFF) for resistance of commensal *E. coli* proposed by the European Committee on Antimicrobial Susceptibility Testing (EUCAST) is 0.03 μg/mL, but was recently adapted to 0.06 μg/mL [2]. Again, intestinal levels are considerably higher than plasma levels and, based on the ECOFF, this would imply a complete eradication of susceptible bacteria.

The present study is the first to describe the enro- and ciprofloxacin concentrations in different parts of the intestinal tract of broiler chickens after conventional and alternative treatment strategies. To assess the possible impact of enrofloxacin treatment on resistance selection in the intestinal microbiota, concentration data in different parts of the intestinal tract, and not only from fecal droppings, is essential. Especially concentrations in cecal and colonal content have to be measured as they harbor the largest amount of intestinal bacteria.

To fully understand the impact of the reported enrofloxacin concentrations on the intestinal microbiota, future research should apply these levels in an *in vitro* gastro-intestinal simulation model or monitor the resistance selection *in vivo* after different treatment strategies. Furthermore, future research might also evaluate the effect of continuous drinking water medication, which is the preferred route of administration in poultry, on intestinal and plasma concentrations of enrofloxacin and compare them with the results of single oral and parental administration presented in this study.

The authors would like to stress out that administration of a fivefold increased dose without reconsidering the duration of the therapy, might lead to improper use of fluoroquinolones. Also, the problem of drug residues and withdrawal times should be considered in such case. When assessing the effect of the different concentrations reported in this study on resistance development or selection, both dose and duration of therapy should be considered. For instance, Haritova et al. (2011) [10] demonstrated a better eradication of pathogenic *E. coli* 078/H12 in broiler chickens with a single oral administration of 50 mg enrofloxacin/kg BW compared to oral administration of 10 mg enrofloxacin/kg BW for 5 consecutive days.

## Conclusions

This is the first paper describing enro- and ciprofloxacin concentrations in content of different parts of the intestinal tract, as well as in plasma, after different treatment strategies of broiler chickens with enrofloxacin. In general, the intestinal microbiota in cecum and colon is exposed to significant levels of enrofloxacin after conventional treatment (21–130 μg/g). A clear increase of intestinal concentrations was demonstrated after administration of a five-fold higher dose (31–454 μg/g). After intramuscular administration, intestinal concentrations were comparable, except for the higher levels in cloaca due to the complete bioavailability and urinary excretion. The impact of the reported intestinal levels on resistance selection in the intestinal microbiota has to be further investigated, e.g. using an *in vitro* gastro-intestinal simulation model or monitor *E. coli* indicator bacteria *in vivo* after different treatment strategies.

## Additional files

Additional file 1:
**Results of the evaluation of linearity (goodness-of-fit coefficient (g), correlation coefficient (r)), limit of quantification (LOQ), limit of detection (LOD) for enrofloxacin (ENRO) and ciprofloxacin (CIPRO) in broiler plasma and pooled intestinal content.**
Additional file 2:
**Results of the within-run and between-run accuracy and precision of enrofloxacin (ENRO) and ciprofloxacin (CIPRO) in plasma and pooled intestinal content from broiler chickens.**
Additional file 3:
**Ratios of the detected concentrations of enrofloxacin in the different intestinal segments (ileum, cecum, colon and cloaca) versus plasma, at 2, 4 and 100 h after the first administration of different doses (10 and 50 mg enrofloxacin/kg body weight) and different administration routes (oral, PO, and intramuscular, IM) of enrofloxacin to broiler chickens.**
