# Supplementary material for: Effect of administration route and dose escalation on plasma and intestinal concentrations of enrofloxacin and ciprofloxacin in broiler chickens
Source: BMC Vet Res. 2014 Dec 2;10:289. doi: 10.1186/s12917-014-0289-1 (PMC4260181; doi:10.1186/s12917-014-0289-1)
Supplement: Additional file 1: — Results of the evaluation of linearity (goodness-of-fit coefficient (g), correlation coefficient (r)), limit of quantification (LOQ), limit of detection (LOD) for enrofloxacin (ENRO) and ciprofloxacin (CIPRO) in broiler plasma and pooled intestinal content. [file 12917_2014_289_MOESM1_ESM.docx]

| **Table S1.** Results of the evaluation of linearity (goodness-of-fit coefficient (g), correlation coefficient (r)), limit of quantification (LOQ), limit of detection (LOD) for enrofloxacin (ENRO) and ciprofloxacin (CIPRO) in broiler plasma and pooled intestinal content | | | | | | |
| --- | --- | --- | --- | --- | --- | --- |
|  |  |  |  |  |  |  |
| Matrix | Analyte | Calibration Range (µg/mL or µg/g) | g  (%) | r | LOQ  (µg/mL or µg/g) | LOD  (µg/mL or µg/g) |
| Plasma | ENRO | 0.05 – 20 | 7.4 | 0.9964 | 0.05 | 0.14 x 10^-3^ |
|  | CIPRO | 0.02 – 20 | 7.7 | 0.9964 | 0.02 | 0.09 x 10^-3^ |
| Pooled intestinal content | ENRO | 0.1 – 20 | 4.3 | 0.9988 | 0.1 | 0.07 x 10^-3^ |
|  | CIPRO | 0.1 – 20 | 8.7 | 0.9956 | 0.1 | 1.19 x 10^-3^ |
|  | | | | | | |
| Acceptance criteria: g ≤ 10 %, r ≥ 0.99 | | | | | | |
