# Supplementary material for: Effect of administration route and dose escalation on plasma and intestinal concentrations of enrofloxacin and ciprofloxacin in broiler chickens
Source: BMC Vet Res. 2014 Dec 2;10:289. doi: 10.1186/s12917-014-0289-1 (PMC4260181; doi:10.1186/s12917-014-0289-1)
Supplement: Additional file 2: — Results of the within-run and between-run accuracy and precision of enrofloxacin (ENRO) and ciprofloxacin (CIPRO) in plasma and pooled intestinal content from broiler chickens. [file 12917_2014_289_MOESM2_ESM.docx]

| Table S2. Results of the within-run and between-run accuracy and precision of enrofloxacin (ENRO) and ciprofloxacin (CIPRO) in plasma and pooled intestinal content from broiler chickens | | | | | |
| --- | --- | --- | --- | --- | --- |
|  | | | | | |
| Matrix | Analyte | Theoretical concentration (µg/mL or µg/g) | Mean concentration ± SD  (µg/mL of µg/g) | Precision  RSD (%) | Accuracy (%) |
| Plasma | ENRO | 0.05^a^ | 0.052 ± 0.002 | 4.5 | 3.7 |
|  |  | 0.1^a^ | 0.097 ± 0.008 | 8.5 | -3.2 |
|  |  | 0.1^b^ | 0.103 ± 0.005 | 4.6 | 2.8 |
|  |  | 1.^a^ | 0.949 ± 0.096 | 10.1 | -5.1 |
|  |  | 1.^b^ | 1.024 ± 0.039 | 3.8 | 2.4 |
|  | CIPRO | 0.02^a^ | 0.021 ± 0.001 | 4.4 | 3.4 |
|  |  | 0.1^a^ | 0.103 ± 0.008 | 7.8 | 2.9 |
|  |  | 0.1^b^ | 0.105 ± 0.010 | 10 | 5.1 |
|  |  | 1^a^ | 1.054 ± 0.039 | 3.7 | 5.4 |
|  |  | 1^b^ | 0.948 ± 0.084 | 8.9 | -5.2 |
| Pooled intestinal content | ENRO | 0.1^a^ | 0.100 ± 0.006 | 5.5 | -0.1 |
|  |  | 0.5^a^ | 0.483 ± 0.015 | 3.1 | -3.4 |
|  |  | 0.5^b^ | 0.502 ± 0.022 | 4.4 | 0.5 |
|  |  | 10^a^ | 10.102 ± 0.308 | 3.1 | 1.0 |
|  |  | 10^b^ | 10.244 ± 0.360 | 3.5 | 2.4 |
|  | CIPRO | 0.1^a^ | 0.100 ± 0.003 | 3.2 | -0.3 |
|  |  | 0.5^a^ | 0.508 ± 0.019 | 3.8 | 1.7 |
|  |  | 0.5^b^ | 0.513 ± 0.022 | 4.4 | 2.6 |
|  |  | 10^a^ | 9.945 ± 0.616 | 6.2 | -0.5 |
|  |  | 10^b^ | 9.928 ± 0.540 | 5.4 | -0.7 |
| ^a^ Within-run accuracy and precision (n=6)  ^b^ Between-run accuracy and precision (n=6); SD: standard deviation; RSD: relative standard deviation;  acceptance criteria: accuracy: -20% to +10%, within-run precision (RSD_max_): 20 ng/mL: 19.2%, 50 ng/mL: 16.7%, 100 ng/mL or ng/g: 15.1%, 500 ng/g: 11.8%, 1000 ng/mL: 10.7%, 10 000 ng/g: 7.5%, between-run precision: 100 ng/mL: 22.6%, 500 ng/g: 17.8%, 1000 ng/mL: 16.0%, 10 000 ng/g: 11.3% | | | | | |
