# Supplementary material for: Effect of administration route and dose escalation on plasma and intestinal concentrations of enrofloxacin and ciprofloxacin in broiler chickens
Source: BMC Vet Res. 2014 Dec 2;10:289. doi: 10.1186/s12917-014-0289-1 (PMC4260181; doi:10.1186/s12917-014-0289-1)
Supplement: Additional file 3: — Ratios of the detected concentrations of enrofloxacin in the different intestinal segments (ileum, cecum, colon and cloaca) versus plasma, at 2, 4 and 100 h after the first administration of different doses (10 and 50 mg enrofloxacin/kg body weight) and different administration routes (oral, PO, and intramuscular, IM) of enrofloxacin to broiler chickens. [file 12917_2014_289_MOESM3_ESM.docx]

| **Table S3.** Ratios of the detected concentrations of enrofloxacin in the different intestinal segments (ileum, cecum, colon and cloaca) versus plasma, at 2, 4 and 100 h after the first administration of different doses (10 and 50 mg enrofloxacin/kg body weight) and different administration routes (oral, PO, and intramuscular, IM) of enrofloxacin to broiler chickens. | | | | | | | | | | | | | | | | |
| --- | --- | --- | --- | --- | --- | --- | --- | --- | --- | --- | --- | --- | --- | --- | --- | --- |
|  | Ileum/plasma | | | Cecum/plasma | | | | | Colon/plasma | | | Cloaca/plasma | | | | |
|  | 2 h | 4 h | 100 h | | 2 h | 4 h | 100 h | 2 h | | 4 h | 100 h | | 2 h | 4 h | 100 h |  |
| 10 mg/kg PO | 15.24 | 21.32 | 23.5 | | 6.98 | 41.64 | 49.31 | 9.89 | | 19.17 | 25.44 | | 6.67 | 13.38 | 21.12 |  |
| 50 mg/kg PO | 23.17 | 19.98 | 20.67 | | 6.99 | 35.23 | 43.96 | 14.1 | | 38.45 | 38.32 | | 6.59 | 29.1 | 34.04 |  |
| 10 mg/kg IM | 2.21 | 4.26 | 8.51 | | 9.57 | 19.04 | 52.6 | 10.01 | | 19.99 | 52.08 | | 14.78 | 28.25 | 66.57 |  |
| 50 mg/kg IM | 3.17 | 5.11 | 11.88 | | 5.29 | 21.44 | 50.23 | 14.7 | | 22.72 | 48.01 | | 21.12 | 29.79 | 65.03 |  |
